# Animal Biomonitoring for the Surveillance of Environment Affected by the Presence of Slight Contamination by β-HCH

**DOI:** 10.3390/antiox11030527

**Published:** 2022-03-10

**Authors:** Alessio Bocedi, Olga Lai, Giada Cattani, Cristina Roncoroni, Giorgia Gambardella, Sara Notari, Francesco Tancredi, Giuseppe Bitonti, Serena Calabrò, Giorgio Ricci

**Affiliations:** 1 Department of Chemical Sciences and Technologies, University of Rome ‘Tor Vergata’, Via della Ricerca Scientifica 1, 00133 Rome, Italy; bcdlss01@uniroma2.it (A.B.); giada.cattani@gmail.com (G.C.); giorgia.gambardella@gmail.com (G.G.); notari_sara@yahoo.it (S.N.); 2 Experimental Zoo-Prophylactic Institute Latium and Tuscany ‘M. Aleandri’, Via Appia Nuova 1411, 00182 Rome, Italy; olga.lai@izslt.it (O.L.); cristina.roncoroni@izslt.it (C.R.); francesco.tancredi@izslt.it (F.T.); giuseppe.bitonti@izslt.it (G.B.); 3 Department of Veterinary Medicine and Animal Production, University of Napoli Federico II, Via Delpino 1, 80137 Napoli, Italy; serena.calabro@unina.it

**Keywords:** environmental pollution, biomarker, erythrocyte glutathione transferase, oxidative stress, exposure assessment, mammal biomonitoring

## Abstract

The aim of this study was to evaluate the influence of hidden environmental pollution on some blood parameters of sheep to detect susceptible biomarkers able to reveal slight contamination. Four dairy sheep farms, two with semi-extensive and two with intensive type systems were involved in this study. Two farms in different systems were chosen as properly located in a southern area of Latium (Italy), close to the Sacco River, in which contamination with β-hexachlorocyclohexane (β-HCH) occurred in the past due to industrial waste. A recent study established the presence of low but detectable residual contamination in these areas. The other two farms were outside the contaminated area. Erythrocyte glutathione transferase (e-GST) and oxidative stress parameters were monitored as well as some immune response and metabolic profile parameters throughout the investigated period of four months. The present study showed a relevant and significant increase in e-GST (+63%) in the extensive farming system of the contaminated area, whereas some immune response biomarkers, i.e., white blood cells, neutrophils, lymphocytes, and lysozyme resulted within the physiological range. In all farms, oxidative stress and acute phase response parameters were also within the physiological range. Our results suggest that e-GST is a very effective alarm signal to reveal “hidden” persistent contamination by β-HCH, and reasonably, by many other different dangerous pollutants.

## 1. Introduction

Environmental pollution represents a planetary emergency due to the development of large industrial areas, increasing car transport, and illegal landfills. Furthermore, the use of pesticides and other agrochemicals and the production every year of many new organic compounds represent another risk because if poured into the soil, water, or air, these may enter the food supply chain or come in direct contact with people [1]. Not all these potentially dangerous compounds can be easily identified, and their impact on the health of humans may be still unclear [2]. Moreover, a few of them may remain silent and persistent in a region either due to their long half-life, or their presence in traces, or their long-term effect on living organisms [3]. Too many times, the presence of toxic compounds in the environment has been revealed belatedly by the unusual frequency of specific pathologies in people or animals. For example, an anomalous incidence of Minamata disease in people and animals revealed contamination by methylmercury in the seawater of Japan [4]. Moreover, a retrospective analysis of the incidence of different tumors in people living in an industrial area of Taranto in Italy revealed a strict correlation with the proximity of an industrial dump [5]. Therefore, the discovery of biomarkers which can promptly reveal the presence of different contaminants that, momentarily, create only a biological alarm response without the onset of co-claimed pathologies may be of great importance [6].

In 2005, due to the detection of β-hexachlorocyclohexane (β-HCH) in a farm’s milk, a southern area of Latium (Italy) near to the Sacco River was in the middle of an environmental emergency [7]. Interestingly, Fabrini and coworkers [8] found an unusually high expression of erythrocyte glutathione transferase (e-GST) in many individuals among five hundred tested people living in this area, suggesting that this enzyme may act as biomarker in humans in case of exposure to toxins, a possibility highlighted in other studies [9,10]. GST is in fact an enzyme devoted to the inactivation and excretion of many toxic compounds from the cell, promoting their conjugation with glutathione [11], and the erythrocyte isoenzyme is hyper-expressed in case of exposure to endogenous or exogenous toxic compounds, a phenomenon likely developed to enhance cellular defenses [12]. Notably, GST synthesis has been found to be triggered by β-HCH exposure [13].

A similar biological response (*Ovis aries*, *Bos taurus*, *Sus scrofa*, *Capra hircus*, *Equus caballus*, and *Equus asinus*) can also be present in other mammalian species as observed by Bocedi and coworkers [14]. Thus, e-GST level was accurately evaluated in this study as well as other well-known biomarkers of contamination. In fact, since pollution may promote the activation of systemic inflammatory responses [15], it could be significant to explore the association of an environmental contamination, even when hidden, with inflammation indicators, oxidative stress, and immune response profiles. In this study, the effect of contamination was assessed on two commercial dairy sheep farms, located in two areas at the extreme border of the low β-HCH contaminated zone with the aim of identifying susceptible biomarkers able to reveal this “borderline” environmental pollution. In this paper the terms “silent contamination” or “hidden environmental pollution” are synonymous and indicate non-massive and non-acute contaminations that do not cause dramatic and immediate health consequences for living organisms.

The two farms had different management systems, since one was an intensive livestock system, using imported and selected forages, whereas the other raised livestock mainly on free pasture in a semi-extensive livestock system. The selection of these farms was made to discriminate whether a possible contamination derived from the soil or from other sources such as water or air. Moreover, as control farms, two commercial dairy sheep farms, also with intensive and semi-extensive livestock systems, were selected in an uncontaminated area. Results indicate e-GST as the main susceptible parameter able to reveal a hidden pollution.

## 2. Materials and Methods

### 2.1. Farms Involved in the Study

**Figure 1:**
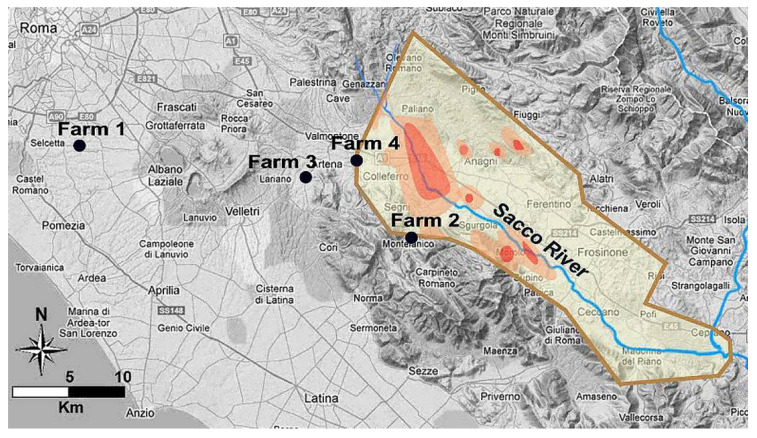
Geospatial map of Latium region (latitude N from 41°26′ to 41°58′ and longitude E from 12°24′ to 13°34′). The map shows the area contaminated by β-HCH (light brown borders) divided into three regions according to different levels of contamination as reported previously [7]: low contamination (yellow), intermediate contamination (orange), and high contamination (red). The map also shows the position of the four farms enrolled in the study (black circles).

Four farms of Latium region (central Italy) were involved in the study. A geospatial map and main characteristics are shown in Figure 1 and Table 1.

Farm 1 (“Reference farm” with a semi-extensive livestock system) close to the Ardeatina district and far from the polluted area, with a total surface area of 400 hectares, reared 4000 heads, especially *Comisana* and *Sarda* breeds, with a medium-low milk production, with livestock mostly grazing natural pastures with a daily supplement of graminaceous hay and pelleted feed.

Farm 2 (“Test farm” with a semi-extensive livestock system) was located at the extreme border of the low contamination zone, near to Montelanico district, with a total surface area of 40 hectares, rearing 300 heads, mostly *Sarda* breed, with a medium-low milk production, with livestock fed daily with grass and legume hay, whole soy, corn, and pelleted feed integrated with cotton.

Farm 3 (“Reference farm” with an intensive livestock system) was near to Giulianello (Artena district), outside the polluted area, rearing 150 *Lacaune* ewes, a recently imported breed with high milk production, fed with *Medicago sativa* hay, barley, ryegrass, soy, cornmeal, beet, and a supplement of proteins.

Farm 4 (“Test farm” with an intensive livestock system) was located at the extreme border of the low contamination zone between Artena and Colleferro district, rearing 200 *Lacaune* heads, fed daily with grass hay, barley, maize, beans, and pelleted feed.

In all farms, animals did not show any critical clinical and veterinary problems.

### 2.2. Blood Samples and Ethics Statement

In the present work, we selected pluriparous and healthy ewes, aged three years (36 ± 4 months), according to the following distribution: Farm 1 (N = 10), Farm 2 (N = 10), Farm 3 (N = 10), and Farm 4 (N = 9). The ewes were tested from the beginning of the milking process in the winter season (2017) until springtime, for 4 months during lactation. Every month peripheral blood samples were collected for each animal from the jugular vein in tubes with K3-EDTA. The animals were injected by a veterinary doctor using an appropriate procedure. The blood samples were kept at 4 °C and delivered to clinical laboratories within 2 h. Moreover, the samples were stored for supplementary analysis at 4 °C for no more than two days. Ethical statements were according to EU Directive 2010/63/EU for animal experiments. Legal and ethical requirements were met with regards to the humane treatment of the animals described in the study. The study was carried out in private farms with permission from the owners. The analyses had the advantage of employing the blood samples collected for routine health controls. Specific permissions from the Italian Ministry of Health (Ordinance n. 153/2001-A) for animal care and use and from Ethics Committee of University of Rome ‘Tor Vergata’ were obtained for this study. The study was carried out on farm animals and did not involve endangered or protected species. None of the animals was sacrificed for this study.

### 2.3. Clinical Analysis and Immunological Parameters

A complete blood cell count was performed using the automated counter Cell-Dyn 3700 (Abbott—12 parameters) (Abbott, Chicago, IL, USA) in order to assess leukocyte (neutrophils and lymphocytes) counts. 

Serum lysozyme (Lyz) activity, a cornerstone of innate immunity, was assessed by microbiologic assay [16]. Haptoglobin (Hp), as a marker of inflammation, was determined in serum samples by commercial ELISA kit (Tridelta Development Ltd., Kildare, Ireland) as previously described [17].The kidney function indicators blood urea nitrogen (BUN) and creatinine (Crea) were determined by an automated analyzer Olympus AU 400 (Beckman, Brea, CA, USA). Oxidative stress was monitored by dRoms test, an assay marketed for analyzing the total amount of hydroperoxides (dROMs) and by OXY Adsorbent Test in order to evaluate the total anti-oxidant action of the plasmatic barrier (OXY) in serum by diagnostic kits (Diacron International, Grosseto, Italy).

### 2.4. Chemicals and Reagents

Glutathione (GSH), 1-chloro-2,4-dinitrobenzene (CDNB), ethylenediaminetetraacetic acid, cystamine, 5,5′-dithiobis(2-nitrobenzoic acid) (DTNB) (Ellman’s reagent), and all other reagents were purchased from Sigma-Aldrich (St. Louis, MO, USA).

### 2.5. Erythrocyte Glutathione Transferase Activity

e-GST activity was measured using a spectrophotometric assay at 340 nm (37 °C) by an Uvikon 941 Plus spectrophotometer (Kontron Instruments, Watford, UK). One volume of 40 μL of whole blood was diluted into 1 mL of bi-distilled water; this first dilution provokes the erythrocytes hemolysis. In a second step, 100 μL of hemolyzed blood was diluted to a final volume of 1 mL of 0.1 M potassium phosphate buffer, pH 6.5, containing 1 mM GSH, 1 mM CDNB, according to Habig and coworkers [18]. Final data were reported as enzyme units (U) per gram of hemoglobin (Hb) (U/g _Hb_) [19]. One unit is the amount of enzyme that catalyzes the conjugation of one micromole of GSH to CDNB in 1 min at 37 °C.

### 2.6. Oxidized Serum Albumin

The percent of oxidized serum albumin (SAox) was determined by subtracting the value of reduced SA from the total SA estimated in clinical analysis. The reduced SA cannot be evaluated directly; the single cysteine (Cys34) not involved in a disulfide bridge reacts very slowly with Ellman’s reagent. A modified procedure based on the reaction of cystamine with Cys34 was adopted [20,21]. The released cysteamine is stoichiometric with Cys34 and can be determined with DTNB (ε_412nm_ of TNBS^−^: 14,100 M^−1^ cm^−1^). The assay was performed on a Kontron Uvikon 941 Plus spectrophotometer (Kontron Instruments, Watford, UK) at 412 nm (25 °C). One volume of 50 μL of serum was diluted in 890 μL of potassium phosphate buffer 0.1 M pH 8.0 recording an autozero and then 50 μL of DTNB (50 μM final concentration) and 10 μL of cystamine (1 mM final concentration) were added to the solution. After ≈ 15 min of incubation the final absorbance was registered.

### 2.7. Statistical and Graphical Analysis

The data analyses of each parameter were reported for each farm as means ± standard deviations (SD). Moreover, to assess the lack of variation during the 4 months with respect to the baseline, each parameter was analyzed in all farms. One-way ANOVA was employed to compare the different dataset for each parameter of ewes deriving from the four farms [22]. Tukey’s multiple comparison test was applied as a post-test; *p* ≤ 0.05 was considered statistically significant [22]. Analysis of correlation was performed to estimate the goodness of fit reported as r^2^ for the parameters e-GST, lymphocyte (LYM), neutrophils (NEU), and white blood cells (WBC). The statistical analysis in the study was performed using the Statistical Package available in GraphPad Prism v8 (San Diego, CA, USA). The graphics and results visualization were obtained by GraphPad Prism v5 (San Diego, CA, USA).

## 3. Results

### 3.1. Farms

In total, four commercial dairy sheep farms were involved in this study. Figure 1 and Table 1 report the location, the livestock system and the feeding method of each farm involved in the study. No clinical or welfare problems were observed.

### 3.2. e-GST, Immune Response, and Infection Biomarkers

Analyses in the four farms were performed monthly for four consecutive months. No correlation and no evident variations (more than 10%) of all hematic parameters were observed throughout the scheduled period of the study (data not shown). As a result, we considered the average values of each biomarker. 

**Figure 2:**
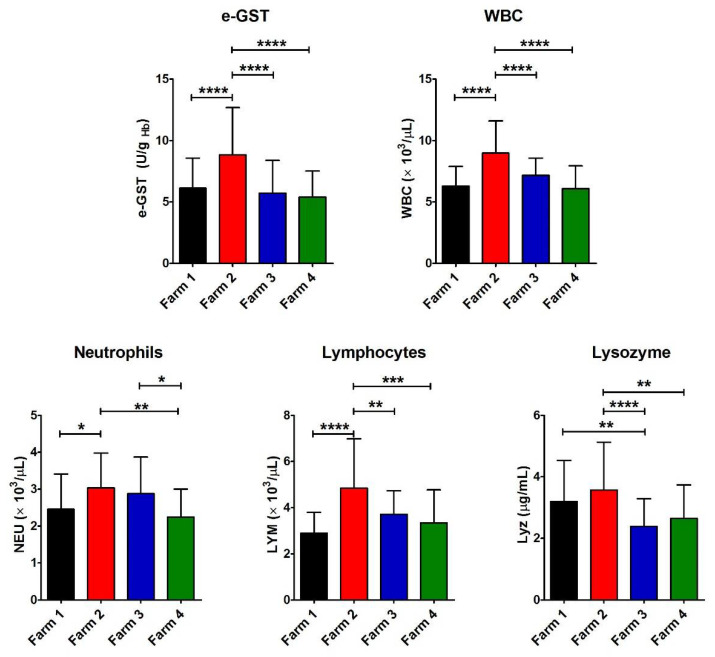
Values of e-GST, immune response, and infection biomarkers. Histograms show the average for the eight parameters measured for each farm (Farm 1–4). The error bars represent the standard deviations. At the top of the columns, the asterisks represent the *P* values (see Materials and Methods) (*p* ≤ 0.05 (*), *p* ≤ 0.01 (**), *p* ≤ 0.001 (***), and *p* ≤ 0.0001 (****)).

In the polluted area, Farm 2, with a semi-extensive livestock system, showed a significant increase in e-GST compared to the other farms, both in a similar polluted area (Farm 4, with an intensive livestock system) and in a not polluted area (Farms 1 and 3 with semi-extensive and intensive livestock systems, respectively) (Figure 2 and Table 2). This enzyme is able to inactivate many toxic compounds by catalyzing their conjugation with glutathione. Its level in erythrocytes increases in case of exposure to many dangerous compounds, and thus it may be considered a long-term indicator of blood toxicity [12]. An increased level of GST was observed in rats contaminated with β-HCH [13]. Consequently, the enhancement of its activity in Farm 2 could be a clear signal of a biological alarm due to a residual contamination.

The Farm 2 exhibited a significant increase in the number of WBC as well, mainly NEU and LYM, although within the physiological range of species (Figure 2 and Table 2). This suggested some incipient bacterial or viral infection, or that the chemical contaminants in this environment were acting similarly to antigens, causing the proliferation of defense cells as previously described for fish living in polluted waters [23]. As a matter of fact, the increase in WBC in mammals often occurs in case of bacterial or viral infection, but not in case of ingestion or exposure to toxic compounds [24].

Nevertheless, there is no correlation with the increase in e-GST, both for WBC (r^2^ = 0.023) and LYM (r^2^ = 0.015) in each head.

Lyz, an enzyme that increases its concentration in case of bacterial attack [16], is significantly higher in both Farm 1 and 2 (both with semi-extensive livestock systems) compared to other farms.

### 3.3. Biomarkers of Kidney Efficiency

**Figure 3:**
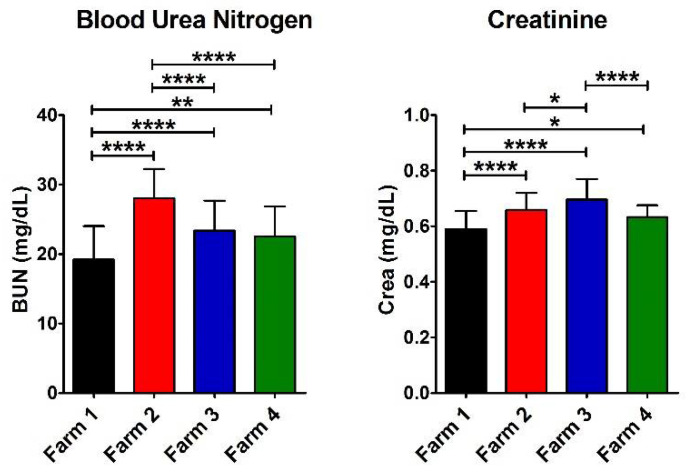
Values of e-GST, immune response, and infection biomarkers. Histograms show the average for the eight parameters measured for each farm (Farm 1–4). The error bars represent the standard deviations. At the top of the columns, the asterisks represent the *p*-values (see Materials and Methods) (*p* ≤ 0.05 (*), *p* ≤ 0.01 (**), and *p* ≤ 0.0001 (****)).

In order to evaluate any possible damage to the kidney efficiency, BUN and Crea were tested on the collected samples in all the farms (Figure 3). A significant increase in BUN was found in Farm 2 compared to Farm 1 and 3, suggesting slight kidney suffering [19,25]. However, the Crea value was within the physiological range of species in all the farms (Table 3). Furthermore, in Farm 1 (with a semi-extensive livestock system and in a not polluted district), we found a significantly lower value compared to all the other farms. The reference values are reported in Table 4.

### 3.4. Biomarkers of Oxidative Stress

**Figure 4:**
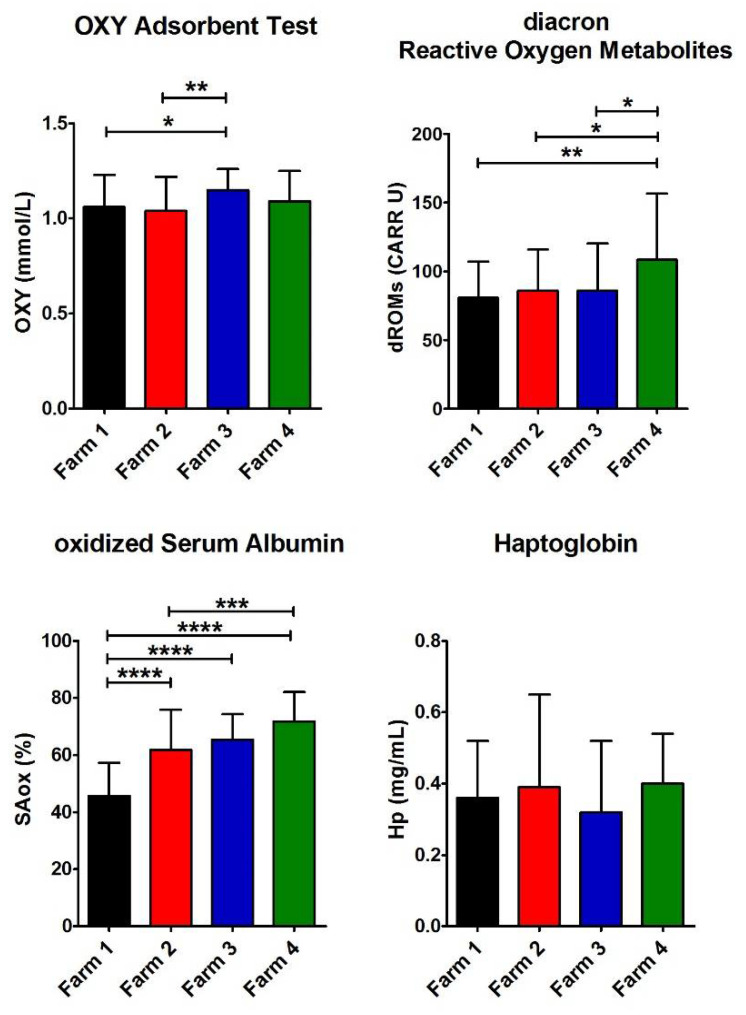
Values of biomarkers of oxidative stress. Histograms show the average for the four parameters measured for each farm (Farm 1–4). The error bars represent the standard deviations. At the top of the columns, the asterisks represent the *p*-values (see Materials and Methods) (*p* ≤ 0.05 (*), *p* ≤ 0.01 (**), *p* ≤ 0.001 (***), and *p* ≤ 0.0001 (****)).

Hp, SAox, dROMs, and OXY were assayed to assess any possible oxidative stress in the farms close to the contaminated area (Table 5 and Figure 4) [32,33,34,35,36]. The reference values are reported in Table 4.

Hp is a marker of inflammation or oxidative stress [37,38,39]. In sheep, Hp increases in response to early production of TNF-alpha in inflammatory reactions [40,41]. The mean Hp level in all the farms was within the normal values reported in healthy ewes (see Table 4) [32,42], but no farm in the contaminated area exhibited higher levels compared to the reference farms. Moreover, Hp in the four farms did not display a significant difference.

Furthermore, in all the farms, the redox status, as the balance between the dROMs and the antioxidant barrier levels, was assessed. dROMs values were close to the upper limit of the physiological range, significantly higher in Farm 4, with an intensive system and in the contaminated area. Moreover, in all the farms, the antioxidant test values are below the reference range, and no relevant differences were found among all the farms.

An additional biomarker of oxidative stress, i.e., the SAox, was also evaluated. Serum albumin is a useful biomarker of oxidative stress because a single thiol group (Cys34) is particularly susceptible to oxidation by reactive oxygen species giving mixed disulfide with cysteine or GSH [21,43].

The highest value of SAox was observed in Farm 4 as well as for dROMs, whereas the lower level was observed in Farm 1 (Table 5 and Figure 4). A common opinion is that contamination always causes oxidative stress [34,36], but these data underline that it cannot occur in case of a low level of pollution.

## 4. Discussion

The presence of environmental pollution in water, air, or fields represents a serious risk both for humans and for animals [7,44,45,46,47,48]. One of the most insidious and dangerous situations in this context is the presence of a “silent and hidden persistent contamination”, i.e., a constant presence of unknown or undetected toxic compounds that, in case of absorption or ingestion by humans or animals, can have dramatic consequences on their health in a medium/long period.

This circumstance occurs frequently in industrialized regions and scarce are the tools to identify early exposure, the chemical nature of contaminants and their concentrations, and the real impact on human health [49,50]. Paradoxically, people can live without evident problems in an area characterized by high levels of toxic compounds if they do not enter in a direct contact with them. On the contrary, very low levels of toxins in the local water or waste may cause dramatic consequences, but their detection may be very difficult and almost impossible before the occurrence of unusual incidences of specific pathologies such as neoplastic diseases. One ideal tool to detect a “hidden contamination” would be effective and specific biomarkers that can reveal the early activation of natural defense mechanisms, such as WBC that usually increase their level in case of hidden infectious diseases. This alarm signal does not characterize the pathogen organism or the site of the infection, but it may represent the first essential indicator, which must be followed by further investigations. The data in this study indicate that e-GST seems to comply with these requirements as its level showed a statistically significant increase in the contaminated Farm 2 with a semi-extensive livestock system. We underline that its enhanced expression in sheep reproduces what was previously observed in hundreds of inhabitants of the Sacco River valley [8]. Thus, e-GST appears to be a useful sensor of contamination for different mammals [14]. Moreover, the ewes of Farm 4, located in the same polluted area, but with an intensive livestock system, show a normal e-GST level. This remarkably points out that the origin of the altered e-GST in Farm 2 should be confined within the free grazing animals, as opposed to the Farm 4 animals, fed with selected and purchased aliments.

A further interesting piece of evidence in Farm 2 is the higher level of WBC and, in particular, the significant increase in NEU (against Farm 1 and 4) and LYM (against Farm 1, 3, and 4). In fact, both these cells are considered to increase in case of infectious organism attacks, by bacteria and viruses, but never in case of toxin exposure or ingestion [24]. Indeed, no correlation has been found between these biomarkers and e-GST, indicating that the sheep in Farm 2 show some slight bacterial sufferance possibly due to their permanence in a free pasture. Moreover, the higher WBC level does not exceed the normal range. They higher value of Lyz in Farm 2 suggests a loss of innate immunity defense, but its value is still within the range of normality. The results obtained by evaluating a few biomarkers of oxidative stress were also surprising. It is common opinion that pollution and exposure to or ingestion of toxic compounds invariably causes oxidative stress [34,36]. In particular, β-HCH is known to trigger this dangerous phenomenon [51]. Our results indicate that the low contamination in Farms 2 and 4 is probably not sufficient to be signaled by several oxidative stress biomarkers (OXY, dROMs, Hp, and SAox). The only remarkable result is the very low level of SAox detected in Farm 1 as a clear signal that semi-extensive farming in the uncontaminated area represents a better livestock system compared to the intensive one.

## 5. Conclusions

Our data indicate that e-GST is the best candidate to reveal “hidden persistent pollution” by β-HCH. However, the expression of this enzyme is stressed by many other toxic compounds, thus it is reasonable that simple screening analysis, performed on human or animals by using just few drops of blood, will help to detect unknown hidden contaminations caused by different pollutants, which will be definitively identified by subsequent chemical investigation. The spectrophotometric analysis for e-GST is very simple, requires only 2 min, and does not need expensive reagents. An important property of this enzyme is that its level remains constant during the erythrocyte life span, so it does not reflect short-term exposure to pollutants but an average time of three–four months, corresponding to the erythrocyte life span in adult sheep. This behaviour remembers the property of glycosylated haemoglobin, which provides a time-average integral of the blood glucose concentration through the 120-day life span of the red blood cell.

## Figures and Tables

**Table 1:** Main characteristics of the examined farms.

Characteristic	Farm 1	Farm 2	Farm 3	Farm 4
**Category of farm**	Reference	Test	Reference	Test
**Type of livestock**	Semi-extensive	Semi-extensive	Intensive	Intensive
**Size of grazing lands**	250 hectares	40 hectares	-	-
**Size of stables**	2300 sqm	400 sqm	500 sqm	450 sqm
**Sheepbreeds**	Comisana and Sarda	Sarda	Lacaune	Lacaune
**No. of heads**	4000	300	150	200
**Sheep feed**	Purchased. Grass hay and pelleted feed (5 h of grazing).	Purchased and company production. Legume hay, grass hay, soybean, corn, pelleted feed with cotton (7 h of grazing).	Purchased and company production. Hay (alfalfa, grass, ryegrass), barley, soy core, cornmeal, beets pulp, protein core.	Purchased and company production. Grass hay, grains (barley, maize, field beans), and pelleted feed.
**Milk production**	Medium-low	Medium-low	High	High
**Company dairy**	No	Yes	Yes	Yes

**Table 2:** Values of e-GST, immune response, and infection biomarkers for the four examined farms.

Parameters	Farm 1	Farm 2	Farm 3	Farm 4
**e-GST (U/g _Hb_)**	6.1 ± 2.4 (50)	8.8 ± 3.9 (50)	5.7 ± 2.7 (40)	5.4 ± 2.1 (35)
**WBC (×10^3^/μL)**	6.3 ± 1.6 (40)	9.0 ± 2.6 (50)	7.2 ± 1.4 (50)	6.1 ± 1.9 (26)
**NEU (×10^3^/μL)**	2.5 ± 1.0 (40)	3.0 ± 0.9 (50)	2.9 ± 1.0 (50)	2.2 ± 0.8 (26)
**LYM (×10^3^/μL)**	2.9 ± 0.9 (40)	4.8 ± 2.1 (50)	3.7 ± 1.0 (50)	3.3 ± 1.4 (26)
**Lyz (µg/mL)**	3.2 ± 1.3 (50)	3.6 ± 1.6 (49)	2.4 ± 0.9 (50)	2.6 ± 1.1 (34)

Data are Mean ± SD (N).

**Table 3:** Values of biomarkers of kidney efficiency measured for the examined farms.

Parameters	Farm 1	Farm 2	Farm 3	Farm 4
**BUN (mg/dL)**	19.2 ± 4.8 (50)	28.0 ± 4.2 (50)	23.4 ± 4.3 (50)	22.5 ± 4.3 (35)
**Crea (mg/dL)**	0.59 ± 0.06 (49)	0.66 ± 0.06 (50)	0.70 ± 0.07 (50)	0.63 ± 0.04 (34)

Data are Mean ± SD (N).

**Table 4:** Reference values of analyzed clinical parameters for sheep.

Parameters	Values	References
Blood Urea Nitrogen (mg/dL)	10–35	[26]
	8–20	[27,28]
Creatinine (mg/dL)	1.2–1.9	[26,27,28]
dROMs (CARR U)	62.80 ± 9.42	[29]
	73.00 ± 3.18	[30]
Haptoglobin (mg/mL)	0.27–0.80	[31]
	0.29 ± 0.17	[17]
Hemoglobin (g/dL)	9.0–15.0	[26,27]
	9.0–14.0	[28]
Lymphocytes (n/μL)	2000–9000	[26,27]
Lysozyme (µg/mL)	1.47 ± 0.71	[17]
Neutrophils (n/μL)	700–6000	[26,27]
OXY (mmol/L)	1.68 ± 0.32	[29]
	2.78 ± 0.11	[30]
White Blood Cells (n/μL)	4000–12,000	[26,27]

**Table 5:** Values of biomarkers of oxidative stress measured for the examined farms.

Parameters	Farm 1	Farm 2	Farm 3	Farm 4
**OXY (mmol/L)**	1.06 ± 0.17 (49)	1.04 ± 0.18 (50)	1.15 ± 0.11 (40)	1.09 ± 0.16 (34)
**dROMs (CARR U)**	80.7 ± 26.4 (40)	85.9 ± 29.9 (50)	86.0 ± 34.2 (50)	108.4 ± 48.2 (26)
**SAox (%)**	45.7 ± 11.6 (50)	61.8 ± 14.1 (50)	65.4 ± 8.9 (40)	71.8 ± 10.3 (34)
**Hp (mg/mL)**	0.36 ± 0.16 (45)	0.39 ± 0.26 (47)	0.32 ± 0.20 (49)	0.40 ± 0.14 (26)

Data are Mean ± SD (N).

## Data Availability

Data is contained within the article.
